# Interspecific plant interaction structures the microbiomes of poplar-soil interface to alter nutrient cycling and utilization

**DOI:** 10.1128/spectrum.03368-23

**Published:** 2024-01-10

**Authors:** Yimin You, Liran Wang, Xiaoting Liu, Xuelai Wang, Luping Jiang, Changjun Ding, Weina Wang, Dawei Zhang, Xiyang Zhao

**Affiliations:** 1 Jilin Provincial Key Laboratory of Tree and Grass Genetics and Breeding, College of Forestry and Grassland Science, Jilin Agricultural University, Changchun, China; 2 National Key Laboratory of Forest Genetics and Breeding, Northeast Forestry University, Harbin, China; 3 Chinese Academy of Forestry, Beijing City, China; 4 Heilongjiang Provincial Forestry Technology Service Center, Heilongjiang Province, China; 5 Fuyu Forest Farm, Qiqihar City, China; Universitetet i Oslo, Oslo, Norway

**Keywords:** intercropping, microbiome, co-occurrence networks, metabolism, rhizosphere

## Abstract

Terrestrial plants can influence the growth and health of adjacent plants through interspecific interaction. Here, the mechanisms of interspecific plant interaction on microbial function and nutrient utilization in the plant-soil interface (non-rhizosphere soil, rhizosphere soil, and root) were studied by soybean- and potato-poplar intercropping. First, metagenomics showed that soybean- and potato-poplar intercropping influenced the composition and co-occurrence networks of microbial communities in different ecological niches, with higher stability of the microbial community in soybean intercropping. Second, the gene abundance related to carbon metabolism, nitrogen cycling, phosphorus cycling, and sulfur cycling was increased at the poplar-soil interface in soybean intercropping. Moreover, soybean intercropping increased soil nutrient content and enzymatic activity. It showed higher metabolic potential in nutrient metabolism and transportation. Third, functional microorganisms that influenced nutrient cycling and transportation in different intercropping have been identified, namely *Acidobacteria*, *Sphingomonas*, *Gemmatimonadaceae*, *Alphaproteobacteria*, and *Bradyrhizobium*. Therefore, intercropping can construct microbial communities to alter metabolic functions and improve nutrient cycling and absorption. Interspecific plant interactions to influence the microbiome were revealed, opening up a new way for the precise regulation of plant microbiome.

**IMPORTANCE:**

Poplar has the characteristics of wide distribution, strong adaptability, and fast growth, which is an ideal tree species for timber forest. In this study, metagenomics and elemental analysis were used to comprehensively reveal the effects of interspecific plant interactions on microbial communities and functions in different ecological niches. It can provide a theoretical basis for the development and application of the precise management model in poplar.

## INTRODUCTION

The intercropping system of agro-forestry refers to the simultaneous planting of at least two plants on a piece of land (1). Intercropping has long been used in many places, including China, Indonesia, India, Niger, Mali, Central America, and Western Europe (2). Over one-third of cassava and bananas grown in the Americas and Africa were intercropped (3). Interspecific plant interactions can produce both positive and negative fitness outcomes. The promotion occurs when the presence of one species changes the environment in a way that improves the fitness of a second neighboring species (4, 5). Certain species can enhance the establishment of their neighbors by improving nutrient availability or microclimate conditions (6). For example, leguminous plants can contribute to higher nitrogen (N) import into other plants via biological N_2_ fixation (7). Promoting effects can occur indirectly through the regulation and activity of soil and plant microorganisms (4, 8). Plant diversity can play a boosting role by reducing soil-borne pathogens (9, 10). Therefore, plants that complement each other will produce higher yields and enhance resource utilization (11). Terrestrial plants tend to accumulate host-specific pathogens over time in their surrounding soil, which ultimately negatively affects plant performance (12).

Subsurface interspecific interactions are potential mechanisms for improving yield and nutrient acquisition through intercropping (13). Studies showed that plants can shape soil microbial communities by root exudations and changes in the element and nutrient content of the soil environment (14). It is known that individual microorganisms related to plants can enhance their ability to acquire nutrients, respond to stress, and inhibit pathogens through microbial metabolic activity and resistance function (2, 15, 16). Therefore, microbial communities at the plant-soil interface (soil, rhizosphere, and roots) have received increasing attention. Currently, progress has been made in understanding the microbiome composition within a single-host habitat. However, little research has been conducted to fully understand the changes in microbiome and function in various ecological niches, including soil, rhizosphere and root.

Soybeans and potatoes are the main food crops in the experimental area. Meanwhile, the use of legumes to provide N to the soil via biological N_2_ fixation is an essential element in enhancing soil nutrients (7). Poplar is one of the fast-growing wood species with the largest cultivated area and the highest timber yield (17). Meanwhile, it is a model organism for the study of perennial woody plants, which can be used as an ideal model for understanding plant-microbial interactions (18, 19). The growth of poplar is partly dependent on the function of the microbial community at the soil-root interface (20). Microorganisms can decompose organic matter, store and release nutrients, and change the transport rate of substances through various biochemical reactions to promote plant growth (21). At present, the research of poplar mainly focused on the single factors of aboveground or underground traits, such as growth, photosynthetic characteristics, and carbon and nitrogen storage (18). However, there is a lack of research on the subsurface nutrient cycling and regulation mechanisms, including the relationship between the microbial community and plant nutrient uptake.

Therefore, poplar was used as a model to understand: (i) How does intercropping alter soil and plant nutrient transportation? (ii) How do microorganisms enhance the nutrient uptake by roots? The ecological niche of the root-soil interface includes soil, rhizosphere and root successively from outside to inside. Rhizodeposit generated from root cap border cells and the rhizodermis provokes a shift in the soil biome. Cellular disjunction of the root surface during lateral root emergence provides a potential entry gate for the rhizosphere microbiota into the root interior (22). Thus, microbial composition will change in different ecological niches (23). In this study, the microbial community and function at the poplar-soil interface, including non-rhizosphere soil, rhizosphere, and poplar root, were characterized by metagenomic sequencing. Furthermore, soil nutrients, plant growth, and enzyme activities were tested to indicate the mechanism of plant diversification on soil microenvironment. These findings help establish a theoretical foundation for the development and application of precise management models in poplar.

## MATERIALS AND METHODS

### Experiment design

The study was conducted in a poplar forest (4 × 4 m) at Fuyu Forest Farm (47°50'N, 124°47'E) in Qiqihar, China. The average annual temperature of the experiment site is −1°C–10°C, and the average annual precipitation is 666 mm. The average elevation is 146 m. The size of each quadrat was 667 m^2^. Three plots were selected for each model. The planting density of clonal poplar was 4 × 4 m, and it was planted for 1 year. These treatments included control, potato (five rows of potato)-poplar (one row of poplar) intercropping, and soybean (five rows of soybean)-poplar (one row of poplar) intercropping. The poplar density of intercropping was the same as that of monoculture (poplar density 4 × 4 m). The row width of soybean and potato was 0.6 m. Soybean was uninoculated *Bradyrhizobium*. The same land preparation, row spacing, fertilization, irrigation, and harvesting procedures were used for 1 year. Half of each intercropping area was occupied by potatoes or soybeans.

The field experiment of intercropping was established in May 2022. The sowing time was May 2022. The application of compound fertilizers (N, P, and K compound) was 120 kg ha^−1^ year^−1^ in all treated. This was consistent with past practice in the region. The method of fertilization was a one-time fertilization of bottom fertilizer at sowing time. All plots were irrigated twice to prevent water scarcity according to the traditional farming methods. The information of past practice and traditional farming methods was based on records and inquiries from tree farms and local growers.

### Sample selection

Soil and plant samples were collected in August 2022 (the end of the growing season). Meanwhile, the ground diameter and tree height of poplar were measured (once in August 2022). The coefficient of variation was calculated with reference to previous methods (24, 25). Non-rhizosphere soil, rhizosphere soil, and poplar roots were collected. Soil samples were collected from the top 20 cm of the soil profile using a soil auger (35 mm diameter). Three plot samples were collected for each processing, and the number of sampling points for each plot was 9, so there were 27 sampling points in total for each processing. Each replica was composed of a mixture of nine samples from the same plot. Non-rhizosphere soil was collected at a distance of 50 cm from poplar. The rhizosphere soil and root sampling steps were as follows: First, the poplar roots were dug out. Second, the large soil and thick layer of soil on the root surface were removed, and the thin layer of soil adhering to the root surface was left behind. Finally, the soil within 2 mm of the root surface was collected, and clean roots were also collected. Poplar roots were distinguished from other plant. The soil within 2 mm of the root and adhering to the root surface is defined as rhizosphere soil. The sample names of various ecological niche in different treatments are shown in Table 1.

**TABLE 1:** The sample names of various ecological niche in different treatments

	Non-rhizosphere soil	Rhizosphere soil	Root
Control	PS_C	RS_C	R_C
Potatoes-poplar	PS_P	RS_P	R_P
Soybean-poplar	PS_S	RS_S	R_S

### Nutrient and enzyme activity determination

NH_4_^+^ and NO_3_^−^ were extracted with 2 M KCl at a soil/extractant ratio of 1:5 after shaking for 60 min at 250 rpm and 25°C (26). Then the extract was filtered through double loop quantitative filter paper (Whatman, China) and was analyzed on a CleverChem ONE spectrophotometer (Alliance company, France) by extraction with KCl solution—automated method with segmented flow analysis (26, 27). Available phosphorus was analyzed by spectrophotometric determination after alkali fusion (28). Total nitrogen, total carbon, total phosphorus, and organic carbon were determined using an element analyzer (ThermoFisher, Germany) (29). Total phosphorus was determined by spectrophotometric determination after alkali fusion (30). The activities of leucine aminopeptidase (LAP) and urease were measured by a soil enzyme activity assay kit (Jianglai Company, China). Leucine aminopeptidase breaks down L-leucine p-nitroaniline to p-nitroaniline. The maximum absorption peak of the product was shown at 450 nm (31). Urea hydrolysis by urease can produce NH_3_-N. It can react with sodium hypochlorite and phenol to produce water-soluble blue dye indophenol blue in a strong alkaline medium. The product has a characteristic absorption peak at 630 nm (32).

### Metagenomic sequencing

#### Extraction and detection of genomic DNA

Total genomic DNA was extracted from soil and root samples using the E.Z.N.A. Soil DNA Kit (Omega Bio-tek, Norcross, GA, U.S.) according to the manufacturer’s instructions. The concentration and purity of extracted DNA were determined with TBS-380 and NanoDrop2000, respectively. DNA extract quality was checked on 1% agarose gel.

#### Library construction and metagenomic sequencing

DNA extract was fragmented to an average size of about 400 bp using Covaris M220 (Gene Company Limited, China) for paired-end library construction. Paired-end library was constructed using NEXTFLEX Rapid DNA-Seq (Bioo Scientific, Austin, TX, USA). Adapters containing the full complement of sequencing primer hybridization sites were ligated to the blunt-end of fragments. Paired-end sequencing was performed on Illumina (Illumina Inc., San Diego, CA, USA) at Majorbio Bio-Pharm Technology Co., Ltd. (Shanghai, China) using HiSeq X Reagent Kits according to the manufacturer’s instructions (www.illumina.com).

The procedure for gene assembly, prediction, and annotation was described in Supplementary Materials and Methods 2.1.

### Data analysis

One-way analysis of variance with a 5% alpha level followed by Tukey’s multiple comparisons test was used to assess the significant differences among all the experimental treatments with IBM SPSS Statistics version 26.0 (Chicago, USA). The significant differences in this study were among control, soybean-, and potato-poplar intercropping. The high-quality sequences were clustered into operational taxonomic units at 97% sequence identity. The microbial taxonomic composition is drawn through R language tools. Among all the treatments, microbial biomarkers were identified by LEfSe (Linear Discriminant Analysis Effect Size) analysis in the online Galaxy interface (http://huttenhower.sph.harvard.edu/galaxy/) (33). Software mothur (version v. 1.30.1; https://mothur.org/wiki/) analysis was used for microbial diversity. The microbial co-occurrence network was analyzed based on inter-family Spearman correlation. The effects of intercropping on the metabolism and function of the microbial community were analyzed by KEGG and COG functional annotation. All results were reported as the mean ± SD.

## RESULTS

### Impacts of intercropping on microbial diversity in different ecological niches at poplar-soil interface

**Fig 1:**
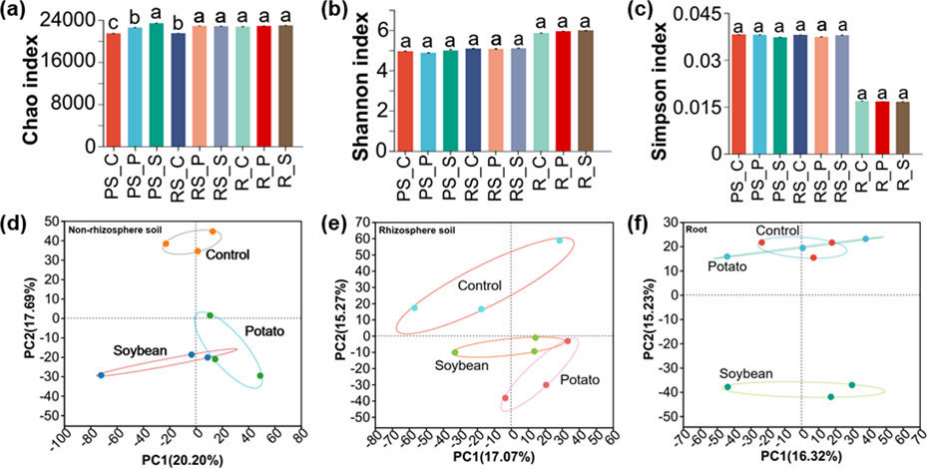
Effects of intercropping on α and β diversity of non-rhizosphere soil (PS), rhizosphere soil (RS) and root (R) microbial communities. (**a**) Chao index, (**b**) Shannon index, and (**c**) Simpson index of microbial community. (**d–f**) PCA analysis (principal component analysis) of microbial species level. In the bar chart, a, b, and c represent the same ecological niche with statistically significant differences between treatments. The three treatments were control group (PS_C/RS_C/R_C), potato intercropping (PS_P/RS_P/R_P), and soybean intercropping (PS_S /RS_S /R_S). On the bar chart, a, b, and c represent significant differences (*P* > 0.05). The significance analysis of the data is presented in the Table S6 to S8.

The impacts of intercropping on the α diversity of the microbial communities at the poplar-soil interface (non-rhizosphere soil, rhizosphere soil, and root) are evaluated. Intercropping increases the microbial richness in non-rhizosphere soil (Chao index), and the trend is the control group (Bulk C) < potato intercropping (Bulk P) < soybean intercropping (Bulk S) (Fig. 1a). In rhizosphere soil, the microbial richness is greater in potato and soybean intercropping than in control group, but there is no difference between the two intercropping (Fig. 1a). There is no difference in the microbial richness within the roots (Fig. 1a). The Shannon index and Simpson index show that intercropping have no significant effect on microbial community diversity in different ecological niches (Fig. 1b and c).

To explore the effects of intercropping on microbial communities, the β diversity analysis of microorganisms is conducted. In PCA, the dispersion distance between treatments demonstrates differences in microbial community composition. It is found that intercropping has different effects on microbial composition in different ecological niches (Fig. 1d through f). In both non-rhizosphere soil and rhizosphere soil, potato and soybean intercropping significantly change the microbial composition (Fig. 1d and e). In the roots, soybean intercropping significantly alters the microbial community, while potato intercropping has no significant effect on it (Fig. 1f).

### Intercropping alters the microbial composition in different ecological niches

**Fig 2:**
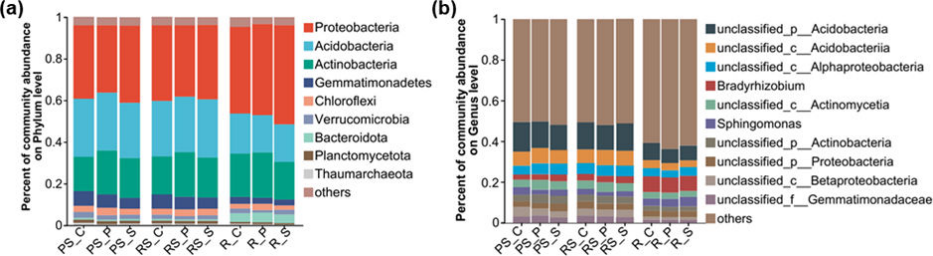
Response of microbial community composition in non-rhizosphere soil (PS), rhizosphere soil (RS), and root (R) to intercropping. (**a**) Microbial community composition at the phyla level. (**b**) Microbial community composition at the genus level. The three treatments were control group (PS_C/RS_C/R_C), potato intercropping (PS_P/RS_P/R_P), and soybean intercropping (PS_S /RS_S /R_S). The significance analysis of microbial phyla is presented in the Table S9 to S11.

In order to study the effect of intercropping on the microbiome, the microbial composition is revealed in different ecological niches at the poplar-soil interface. At the phylum level, the relative abundance of *Proteobacteria* (32%–46%), *Acidobacterium* (17%–27%), *Actinobacteria* (16%–21%), *Gemmatimonadetes* (2%–7%), and *Chloroflexi* (1%–3%) in all sample account for over 80% of the total sequence was classified as dominant (Fig. 2a). In non-rhizosphere soil, the relative abundance of *Proteobacteria*, *Gemmatimonadetes*, and *Verrucomicrobia* in the potato intercropping is decreased compared to the control, while the relative abundance of *Actinobacteria* and *Chloroflexi* is increased (Fig. 2a). Soybean intercropping decreases the relative abundance of *Gemmatimonadetes* and *Verrucomicrobia* and increases the relative abundance of *Proteobacteria* and *Actinobacteria* in non-rhizosphere soil (Fig. 2a). The effects of intercropping on microbial composition in rhizosphere soil are similar to those in non-rhizosphere soil (Fig. 2a). In the root, the relative abundance of *Acidobacterium*, *Gemmatimonadetes*, and *Verrucomicrobia* is reduced in the potato intercropping compared to the control, while the relative abundance of *Chloroflexi* and *Bacteroidota* is increased. Soybean intercropping significantly reduces the relative abundance of *Actinobacteria* and *Gemmatimonadetes* while increasing the relative abundance of *Proteobacteri* in root (Fig. 2a).

At the genus level, the dominant genera are similar in non-rhizosphere soil, rhizosphere soil, and roots of three treatments. However, both potato and soybean intercropping have significant effects on the relative abundance of the microbial community (Fig. 2b). This study uses LEfSe analysis to identify significantly different genera of bacteria, fungi, and archaea, respectively (Fig. S1). In non-rhizosphere soils, the most significant biomarker taxonomic units (MSBT) are the *Afipia*, *Gemmatirosa*, *Microlunatus*, and *Microbotryum* genera of the control group; the *Abiotrophia*, *Umbelopsis*, *Aciduliprofundum*, and *Methanobacterium* genera of the potato intercropping; the *Edaphobacter*, *Nakazawaea*, and *Lepdopterella*genera of soybean intercropping (Figure S1a through S1c). In the rhizosphere soil, the MSBT in the control group is *Pseudolabrys* and *Corynespora* genera; in potato intercropping, the *Sulfopaludibacter* and *Corynespora* genera are the MSBT; in soybean intercropping, the *Altererythriobacter* genera are the MSBT (Fig. S1d and S1e). In the root, the MSBT in the control group is *Enterococcus* genera. The MSBT is *Intrasporangium* and *Gigaspora* genera in the potato intercropping; and it is *Altererythriobacter*, *Porphyrobacter*, *Novosphingobium*, and *Paraphoma* genera in the soybean intercropping (Fig. S1f through S1h). These results suggest that intercropping can alter and construct the microbiome of different ecological niches at the poplar-soil interface.

### Co-occurrence networks of microbiomes in different ecological niches

**Fig 3:**
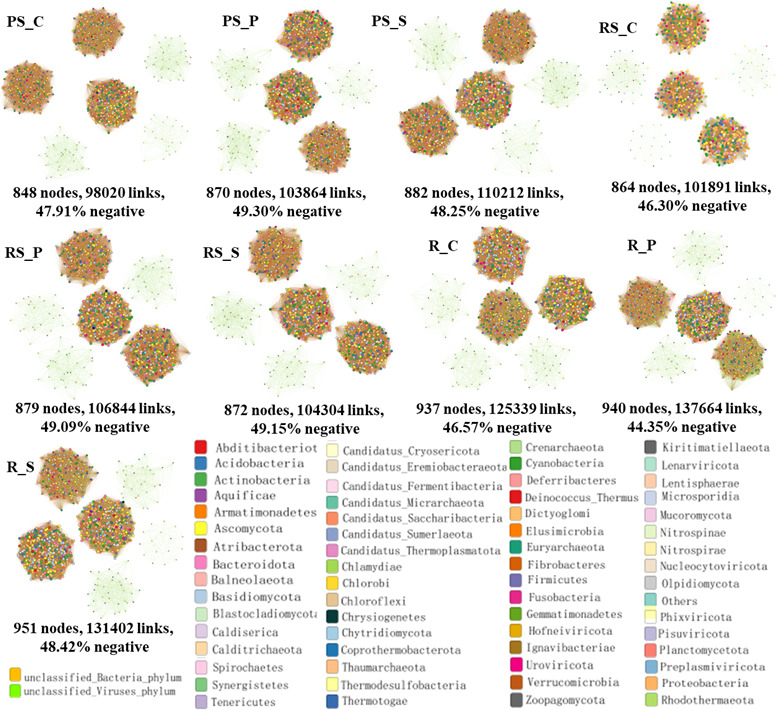
Co-occurrence network of microbial community in non-rhizosphere soil (PS), rhizosphere soil (RS), and root (R). The size of the nodes in the graph represents the abundance of the species, where the larger the abundance value, the larger the node. The color of the line represents a positive and negative correlation. Red indicates a positive correlation between species, while green indicates a negative correlation between species. The thickness of the line represents the magnitude of the correlation coefficient value. A thick line indicates a high correlation between species. A large number of lines indicates a close relationship between this species and other species. The three treatments were control group (PS_C/RS_C/R_C), potato intercropping (PS_P/RS_P/R_P), and soybean intercropping (PS_S /RS_S /R_S). The significance analysis of the data is presented in the supplementary table S12 to S14.

The interaction of microbial communities in different ecological niches at the poplar-soil interface is studied (Fig. 3; Fig. S2 to S4). Topological features of the microbial community are calculated to decipher complex co-occurrence patterns (Fig. 3; Table S1). The mean clustering coefficient and mean path distance (GD) show no difference (Table S1). The total nodes, total links, and average degree (avgK) of microbial network in non-rhizosphere soil are control group < potato intercropping < soybean intercropping (Fig. 3; Table S1). The negative correlation ratio is control group < soybean intercropping < potato intercropping. The total nodes, total links, and avgK of the microbial network in the rhizosphere soil are the control group < soybean intercropping < potato intercropping. The negative correlation ratio is control < potato intercropping < soybean intercropping (Fig. 3; Table S1). The total nodes of the microbial network in the root are the control group < potato intercropping < soybean intercropping, while the total links and avgK are the control group < soybean intercropping < potato intercropping (Fig. 3; Table S1). The negative correlation ratio is potato intercropping < control group < soybean intercropping. There is no significant change in Modularity in non-rhizosphere and rhizosphere soil, while the potato intercropping is smaller than the control and soybean intercropping in root (Fig. 3; Table S1). The links, nodes, and topological features of bacterial, eukaryotic, and archaeal networks are also affected by intercropping. The specific changes are shown in Fig. S2 to S4; Table S2 to S4.

### Effects of intercropping on microbial nutrient metabolism and transport function

**Fig 4:**
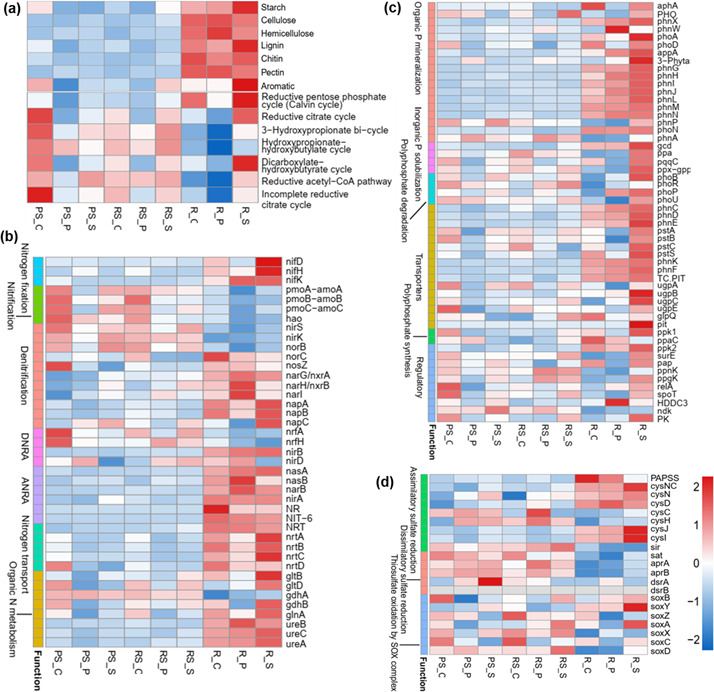
Effects of intercropping on nutrient metabolism and transport pathways of microbial communities in different niches at the root-soil interface. (**a**) Carbon decomposition and fixation, (**b**) nitrogen cycling, (**c**) phosphorus cycling, and (**d**) sulfur cycling. The colors on the side of graphs (**b**), (**c**), and (**d**) show the different metabolic pathways. The colors on the heat map represent changes in gene abundance. The number scale is a value of normalized gene abundance and is used to characterize the size of the gene abundance. The three treatments were control group (PS_C/RS_C/R_C), potato intercropping (PS_P/RS_P/R_P), and soybean intercropping (PS_S /RS_S /R_S).

The effects of intercropping on the metabolism and function of the microbial community are analyzed. The annotated metabolic pathways and functions of all samples are similar (Fig. S5), but there are significant differences in detailed functions. This study focuses on pathways related to nutrient metabolism and transportation. In the three niches, there is no effect on the total abundance of genes associated with carbon decomposition (Fig. S6a). However, the gene abundance of different substances metabolism has been altered, including starch, lignin hemicellulose, cellulose, pectin, chitin, and aromatic. For example, in non-rhizosphere soil, soybean intercropping increased the gene abundance of cellulose and hemicellulose metabolism. Potato intercropping increases the gene abundance of hemicellulose and pectin metabolism (Fig. 4a). Potato intercropping significantly reduces the abundance of carbon fixation-related genes in different ecological niches (Fig. 4a; Fig. S6b). Soybean intercropping increases the abundance of genes related to the reductive acetyl CoA pathway in non-rhizosphere soil. Moreover, it significantly increases the gene abundance related to carbon fixation in both rhizosphere soil and root (Fig. 4a).

This study finds that intercropping also alters the abundance of nitrogen cycling-related genes at the poplar-soil interface. Potato intercropping significantly reduces the total gene abundance of nitrogen cycling, including nitrification and nitrogen transport (Fig. 4b; Fig. S6c). Soybean intercropping significantly increases the total abundance of genes related to nitrogen cycling in the root, including nitrogen fixation, nitrogen transport, and organic N metabolism (Fig. 4b; Fig. S6c). The effects of intercropping on phosphorus cycling are similar to nitrogen cycling (Fig. 4c; Fig. S6d). Overall, potato intercropping significantly decreases the gene abundance of inorganic P solubilization (*gcd*, *ppa*, *pqqC*, and *ppx−gppA* genes) and transporters (*pstA*, *pstB*, *pstC*, and *pstS* genes) in the three ecological niches (Fig. 4c). However, soybean intercropping is significantly increased by soybean intercropping in the root. The total gene abundance of sulfur cycling in non-rhizosphere soil is reduced in potato intercropping, especially in thiosulfate oxidation by SOX complex (*soxX* and *soxC* genes; Fig. 4d; Fig. S6e). Soybean intercropping increases the total gene abundance of sulfur cycling in the non-rhizosphere soil and root (Fig. 4d; Fig. S6e).

### Intercropping alters nutrient content at the plant-soil interface

**Fig 5:**
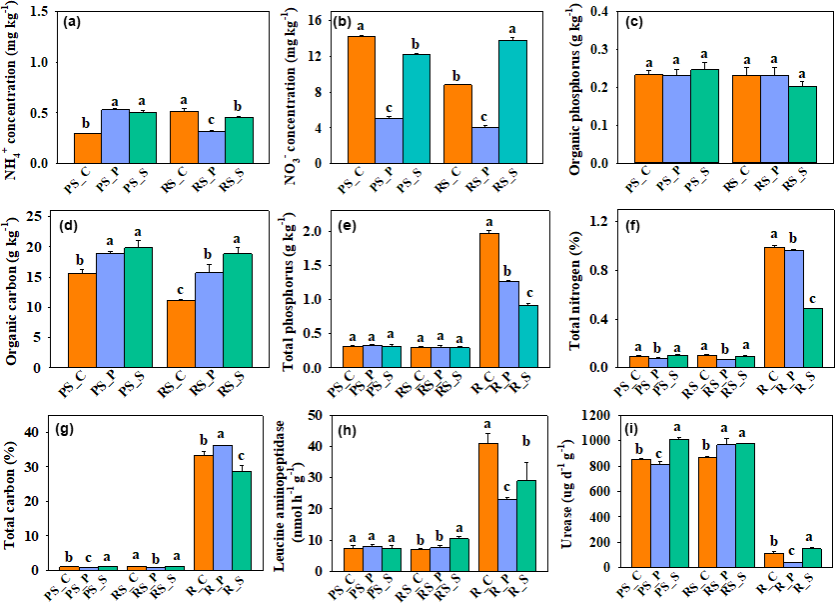
Nutrient content and enzyme activity of non-rhizosphere soil, rhizosphere soil, and root. The three treatments were control group (PS_C/RS_C/R_C), potato intercropping (PS_P/RS_P/R_P), and soybean intercropping (PS_S /RS_S /R_S). On the bar chart, a, b, and c represent significant differences (*P* > 0.05). The data are mean and SD, and the sample number is 5 (*n* = 5). The significance analysis of the data is presented in the Table S15 to S17.

As the analysis of microbial metabolism and function shows that intercropping affects nutrient metabolism and transport of microorganisms in different ecological niches at the poplar-soil interface, nutrient content and enzyme activity in different ecological niches are measured. In non-rhizosphere soil, organic carbon is increased significantly in both intercropping, and the increase is even greater in soybean intercropping (Fig. 5d). NH_4_^+^ is increased significantly in both intercropping, and NO_3_^−^ is also changed (Fig. 5a and b). The total nitrogen in potato intercropping is significantly reduced (Fig. 5f). Soybean intercropping significantly increases the total carbon and urease, which are significantly decreased in potato intercropping (Fig. 5g and i). The NH_4_^+^ and NO_3_^−^ are decreased significantly in rhizosphere soil, and the effect of potato intercropping is more significant (Fig. 5a and b). Potato intercropping significantly reduces total carbon and total nitrogen (Fig. 5f and g). Leucine aminopeptidase is increased significantly in soybean intercropping. Urease is increased significantly in both intercropping (Fig. 5h). In the roots, both intercropping significantly decrease the total phosphorus (Fig. 5e). Soybean intercropping significantly decreases total nitrogen and total carbon, while potato intercropping significantly increases total carbon (Fig. 5f and g). The change of leucine aminopeptidase and urease is altered (Fig. 5h and i). The comprehensive analysis shows that soybean intercropping increases nitrogen and carbon content in soil and significantly decreases total phosphorus, total carbon, and total nitrogen content in roots. Potato intercropping decreases nitrogen content in soil and phosphorus content in roots and increases carbon content in soil and root.

In addition, we analyze the coefficient of variation by ground diameter and height of poplar to reveal the effect of intercropping on poplar growth. The height of poplar is increased in soybean intercropping, but it is not significant (Table S5). The coefficient of variation for tree height and ground diameter is 10.25% and 19.25%, respectively (Table S5).

### Analysis of microorganisms controlling nutrient cycling

**Fig 6:**
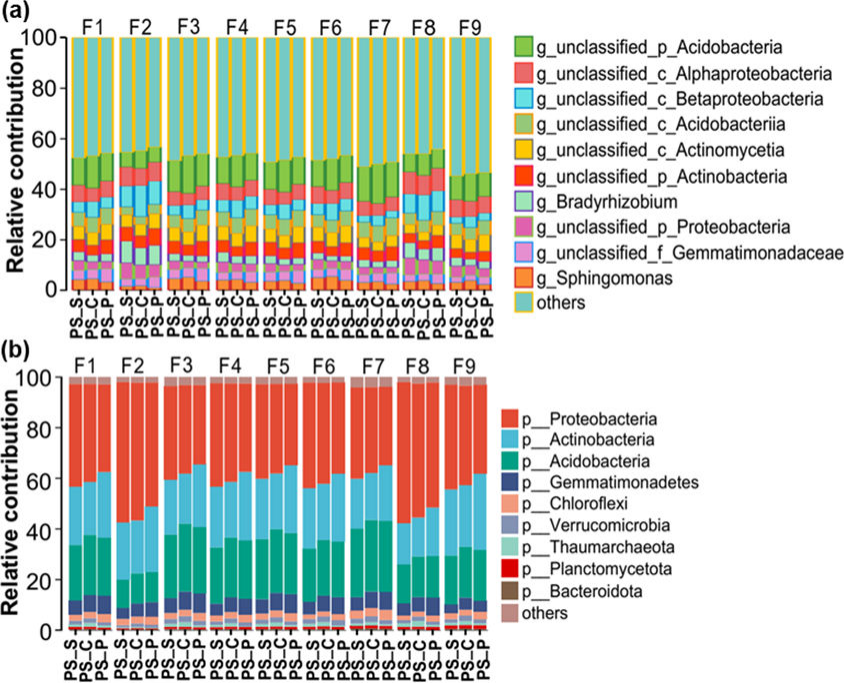
Species and functional contributions of genus (**a**) and phylum level (**b**) in non-rhizosphere soils. F1 Carbon metabolism, F2 ABC transporters, F3 Oxidative phosphorylation, F4 Pyruvate metabolism, F5 Glycolysis/Gluconeogenesis, F6 TCA cycle, F7 Pentose phosphate pathway, F8 Sulfur metabolism, and F9 Nitrogen metabolism. The three treatments were control group (PS_C), potato intercropping (PS_P), and soybean intercropping (PS_S).

The contribution degree of microorganism to nutrient metabolism and transport pathways is analyzed by species and function contribution degree. In non-rhizosphere soils, carbon metabolism, oxidative phosphorylation, pyruvate metabolism, glycolysis/gluconeogenesis, and TCA (tricarboxylic acid) cycle are mainly operated by *Acidobacteria*, followed by *Alphaproteobacteria*, *Actinobacteria*, and *Sphingomonas* (Fig. 6a). In the rhizosphere soil, carbon metabolism, oxidative photosynthesis, pyruvate metabolism, glycolysis/gluconeogenesis, and TCA cycle are affected by *Acidobacteria*, *Alphaproteobacteria*, *Acidobacteriia*, and *Gemmatimonadaceae* (Fig. S7a). The microorganisms that influence ABC transporters, nitrogen metabolism, and sulfur metabolism are similar in non-rhizosphere and rhizosphere soils. ABC transporters and sulfur metabolism are mainly influenced by *Alphaproteobacteria*, followed by *Betaproteobacteria*, *Alphaproteobacteria*, and *Bradyrhizobium* (Fig. 6A; Fig. S7a). In the root, carbon metabolism, pyruvate metabolism, glycolysis/gluconeogenesis, TCA cycle, and nitrogen metabolism are affected by *Bradyrhizobium* and *Acidobacteria*, followed by *Alphaproteobacteria* and *Sphingomonas* (Fig. S8a). ABC transporters and sulfur metabolism are influenced by *Bradyrhizobium*, *Alphaproteobacteria*, *Sphingomonas*, and *Mesorhizobium*. Oxidative phosphorylation is mainly influenced by *Acidobacteria* and *Sphingomonas* (Fig. S8a). The microbial communities that influence carbon metabolism, pyruvate metabolism, glycolysis/gluconeogenesis, and TCA cycle are similar, while the control ABC transporters and sulfur metabolism are similar. In addition, the analysis of microbial and functional contribution at the phylum level also finds that nutrient metabolic pathways are influenced by different phyla (Fig. 6B; Fig. S7b and S8b). Therefore, these results indicate that different microbial communities may play different roles in ecosystems, especially *Acidobacteria*, *Alphaproteobacteria*, and *Sphingomonas*.

## DISCUSSION

The importance of intercropping practices aimed at promoting sustainable production and suppressing diseases has long been recognized in agricultural practice and forestry (3). However, the functional mechanism of microbiome in various ecological niches (soil to root) at the plant-soil interface has not been effectively tested in agroforestry intercropping. Here, our results support the hypothesis that intercropping can change the composition and co-occurrence networks of microbial communities at different ecological niches to influence carbon metabolism, nitrogen cycling, phosphorus cycling, and sulfur cycling. This may occur by altering interspecific interactions and plant root secretions (14). These results suggested that the ability of plants to recruit root-beneficial microorganisms can be activated by the presence of specific neighboring plants (34). This study provides new avenues for research by illustrating that harnessing the plant microbiome for beneficial outcomes can benefit from exploiting plant interspecific interactions with crop designs and their responses in natural and managed ecosystems.

### The interspecific plant interaction influenced the microbial composition

The research on plant diversity has a long history and focuses on interspecific interactions and their residual effects on soil (35, 36). This study explores the effects of terrestrial plants on the growth and health of neighboring plants through interspecies interactions. Soybean- and potato-poplar intercropping change the microbial community composition of different ecological niches (non-rhizosphere soil, rhizosphere soil, and poplar root) at the poplar-soil interface. We find that intercropping increases the microbial richness in both non-rhizosphere and rhizosphere soils, especially soybean intercropping (Fig. 1a). It has no significant impact on the microbial diversity in roots (Fig. 1b and c). This finding is consistent with previous observations that intercropping did not necessarily increase the diversity of microorganisms (37). It was worth noting that the microbial diversity of roots was the highest in different ecological niches. Although the selection intensity varies between microbiomes, the reason for differences in the root and soil microbiome may be the selection of unique microorganisms by poplar, which have the ability to penetrate and survive in the host environment (38). The β diversity and microbial community indicate that soybean intercropping and potato intercropping change the microbial composition at the poplar-soil interface, with a greater impact of soybean intercropping. In contrast to monoculture systems, soil properties were stimulated and regulated by beneficial microorganisms from interplants (39, 40). Soybean intercropping has a greater impact on the microbial communities in non-rhizosphere soil, rhizosphere soil, and roots, indicating that soybean intercropping may have greater functional potential. The reason may be that soybean can increase soil nitrogen through nitrogen fixation of rhizobium (41). These results suggest that the beneficial promoting effect of intercropping is achieved through the dynamic regulation of microbial communities in soil, rhizosphere, and root (Fig. 1d through f). The results of functional annotation also supported the idea that soil microbial communities mediated plant diversity-productivity relationships to some extent (39, 42).

The microbial networks are changed with intercropping in non-rhizosphere soil, rhizosphere soil, and roots. It indicates that the microbial interactions are altered (43). It can be seen that the nodes, links, and negative correlation ratio of microorganisms in non-rhizosphere soil and rhizosphere soil are increased in potato intercropping, while the negative correlation ratio of microorganisms in roots is decreased. Soybean intercropping increases the nodes, connections, and negative correlations ratio of microorganisms in non-rhizosphere soil, rhizosphere soil, and root. Thus, these suggest significant differences in microbial networks at the poplar-soil interface between control, potato intercropping, and soybean intercropping. The three ecological niches show that soybean intercropping improves the microbial stability of poplar-soil interface (Fig. 3). This finding was supported by previous observations that more negative correlations improved the stability of microbial communities (44, 45). Therefore, the microbial network at the poplar-soil interface in the soybean-poplar intercropping may be more stable. This suggests that the ability to maintain interspecific relationships, species numbers, and resistance to stress could be enhanced.

### Intercropping changed the nutrient cycling and transport at the plant-soil interface

Changes in microbial communities will directly affect nutrient cycling in forest ecosystems, including carbon cycling and nitrogen conversion (23). Soybean interplanting increases the gene abundance related to carbon metabolism, nitrogen cycling, phosphorus cycling, and sulfur cycling in different ecological niches at the poplar-soil interface, including carbon fixation, nitrogen fixation, nitrogen transport, inorganic P solubilization, polyphosphate degradation, and transporters (Fig. 4). The results of urease and leucine aminopeptidase activities in non-rhizosphere soil and rhizosphere soil also prove that soybean intercropping promotes nitrogen utilization (46). Meanwhile, soybean intercropping increases soil nitrogen and carbon content, while potato intercropping decreases soil nitrogen (Fig. 5). This is consistent with previous studies, which found that soybean can change soil nutrients by fixing atmospheric nitrogen with compatible microorganism (47, 48). Potato requires large amounts of fertilizer, so they need to absorb nitrogen from the soil (49). In addition, legumes promoted biological nitrogen fixation to enhance soil nitrogen and carbon storage (50). In this study, soybean intercropping reduces nutrient content in roots, which may be attributed to accelerated nutrient metabolism and transport for plant growth. Therefore, the comprehensive results of gene, enzyme, and nutrient content proved that the microbiome of different ecological niches at the poplar-soil interface showed higher metabolic potential in nutrient metabolism and transportation in soybean intercropping. Moreover, soybean-poplar intercropping may increase soil nutrient content and promote plant nutrient utilization.

The microbial community has a dynamic impact on plant growth, health, and performance (14). Plants dynamically respond to environmental changes through changes in physiological and morphological characteristics (i.e., phenotypic plasticity), resulting in direct adaptive benefits (51). Previous studies have shown that the coefficient of variation is greater than 10%, indicating that variation has occurred (52). The variation coefficient (potato intercropping 10.25% and soybean intercropping 19.25%) in this study indicates that intercropping leads to differences in poplar growth (Table S5). Therefore, soybean intercropping is more suitable for agroforestry with poplar.

### Functional microbiome influenced nutrient cycling and transport

The microbiome plays an important role in promoting the utilization of nutrients by plants (16). The contribution degree of microbial function indicates that there are differences in the contributions of different microorganisms to nutrient metabolism, energy metabolism, and transportation pathways (Fig. 6; Fig. S7 and S8). Carbon metabolism, nitrogen metabolism, sulfur metabolism, oxidative phosphorylation, pyruvate metabolism, glycolysis/gluconeogenesis, TCA cycle, and ABC transporters in different ecological niches of poplar-soil interface are mainly affected by *Acidobacteria*, *Sphingomonas*, *Gemmatimonadaceae*, *Alphaproteobacteria*, and *Bradyrhizobium* (Fig. 6; Fig. S7 and S8). Previous studies showed that *Proteobacteria* can be used to measure soil nutrient levels, and fertile soil was conducive to *Proteobacteria* reproduction (53, 54). This phylum participates in the sulfur and nitrogen cycles. Meanwhile, most denitrifying bacteria are *Proteobacteria* (54). Therefore, increasing the relative abundance of *Proteobacteria* by soybean intercropping may promote the metabolism of nitrogen in soil. *Acidobacterium* is associated with Fe in roots, which is consistent with several studies reported. *Acidobacterium* is a rhizosphere colonizer and can produce ferriferous carriers (55). *Bradyrhizobium* has a symbiotic relationship with leguminous plants and participates in nitrogen fixation (56). *Gemmatimonadaceae* and *Sphingomonas* are known plant-promoting microorganisms, which can dissolve phosphorus, sulfur, and calcium and produce a variety of plant hormones and iron carriers (57, 58). Changes in *Sphingomonas*, a rhizosphere biomarker, could prove that the poplar rhizosphere provides sufficient air and organic nutrient conditions for these microorganisms (57). It has been demonstrated that microbial communities isolated from Populus can enhance the health, growth, and development of plant hosts (43, 46). These results indicate that agroforestry could change the nutrient metabolism, energy metabolism, and transport function of the microbiome in different ecological niches of poplar-soil interface by influencing the microbial composition (Fig. 6; Fig. S7 and S8).

Overall, the introduction of functional plants for agroforestry can alter soil chemical properties (nitrogen and phosphorus concentrations) and root properties (nitrogen-fixing bacteria and mycorrhizal fungi) (59–61). These factors may regulate the structural and functional properties of soil microbiome, which further affects the formation and storage of NO_3_^−^, NH_4_^+^, and organic carbon (62, 63). The differences in these functional traits have a significant impact on the functionality of forest ecosystems, including improving the rate of nutrient cycling and transportation (64, 65). Therefore, it is particularly important to explore suitable management models and build a composite ecosystem for tree cultivation by intercropping (2).

## CONCLUSION

**Fig 7:**
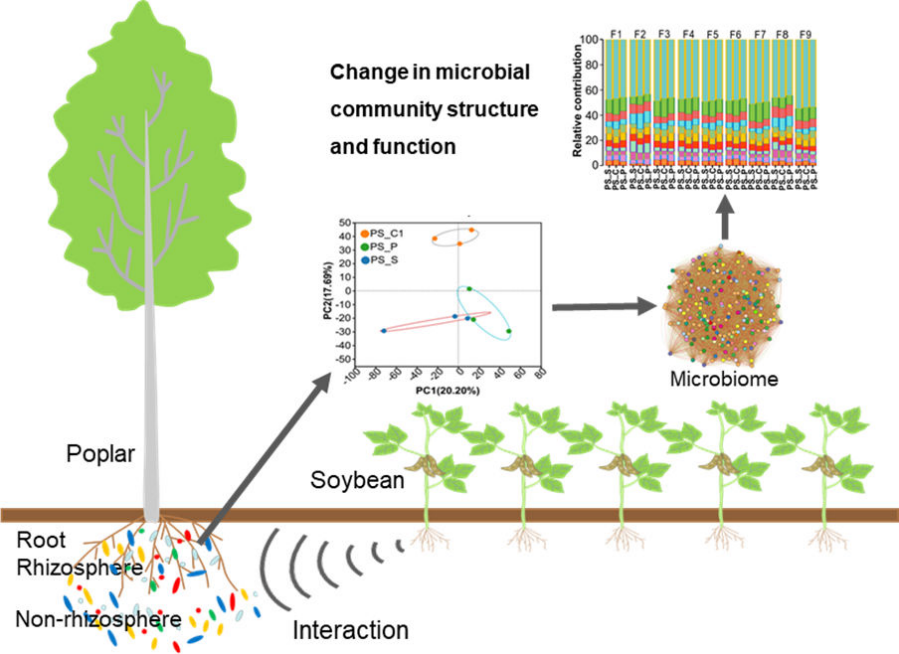
Agroforestry changes the structure and function of microorganisms at the root-soil interface.

Interspecific plant interactions can produce both positive and negative fitness outcomes. This study provides theoretical support for the principles of interspecific plant interactions. This study showed that intercropping influenced the microbial composition, co-occurrence networks, and nutrient cycling in different ecological niches (non-rhizosphere soil, rhizosphere soil, and root) at the poplar-soil interface (Fig. 7). Specifically, soybean intercropping increased soil nutrient content, which promoted soil fertility maintenance and plant nutrient utilization. This can provide a technical model for regulating soil nutrients in forest land and improving the economic value of artificial forests. We identified microorganisms that influenced nutrient cycling and transportation functions in different intercropping, including *Acidobacteria*, *Sphingomonas*, *Gemmatimonadaceae*, *Alphaproteobacteria*, and *Bradyrhizobium*. These results indicated that intercropping can improve soil nutrient cycling and plant nutrient utilization by manipulating microbial communities to alter metabolic functions. This may further understand the influence of the microbial community on ecosystem function. It is necessary to further study the regulatory mechanism of interspecific plant interaction in order to maximize the technical advantages of interspecific system.

## Data Availability

Sequence data associated with this project have been deposited in the NCBI under accession number SRP445747.

## SUPPLEMENTAL MATERIAL

Supplemental material spectrum.03368-23-s0001.docx Supplemental methods, Fig. S1 to S8, and Tables S1 to 17.

ASM does not own the copyrights to Supplemental Material that may be linked to, or accessed through, an article. The authors have granted ASM a non-exclusive, world-wide license to publish the Supplemental Material files. Please contact the corresponding author directly for reuse.
