# Supplementary material for: Interspecific plant interaction structures the microbiomes of poplar-soil interface to alter nutrient cycling and utilization
Source: Microbiol Spectr. 2024 Jan 10;12(2):e03368-23. doi: 10.1128/spectrum.03368-23 (PMC10846221; doi:10.1128/spectrum.03368-23)
Supplement: Supplemental material — Supplemental methods, Fig. S1 to S8, and Tables S1 to 17. [file spectrum.03368-23-s0001.docx]

**2.1 Supplementary materials and methods**

The data were analyzed on the free online platform of Majorbio Cloud Platform (www.majorbio.com). Briefly, the paired-end Illumina reads were trimmed of adaptors, and low-quality reads (length<50 bp or with a quality value <20 or having N bases) were removed by fastp (https://github.com/OpenGene/fastp, version 0.20.0).

Open reading frames (ORFs) from each assembled contig were predicted using Prodigal/MetaGene (http://metagene.cb.k.u-tokyo.ac.jp/). The predicted ORFs with a length ≥ 100 bp were retrieved and translated into amino acid sequences using the NCBI translation table (http://www.ncbi.nlm.nih.gov/Taxonomy/taxonomyhome.html/index.cgi?chapter=tgencodes#SG1.

A non-redundant gene catalog was constructed using CD-HIT (http://www.bioinformatics.org/cd-hit/, version 4.6.1) with 90% sequence identity and 90% coverage. High-quality reads were aligned to the non-redundant gene catalogs to calculate gene abundance with 95% identity using SOAPaligner (http://soap.genomics.org.cn/, version 2.21).

[Representative sequences](http://en.wikipedia.org/wiki/Representative_sequences) of non-redundant gene catalog were aligned to NR database with an e-value cutoff of 1e-5 using Diamond (http://www.diamondsearch.org/index.php, version 0.8.35) for taxonomic annotations. Cluster of orthologous groups of proteins (COG) annotation for the [representative sequences](http://en.wikipedia.org/wiki/Representative_sequences) was performed using Diamond (http://www.diamondsearch.org/index.php, version 0.8.35) against eggNOG database with an e-value cutoff of 1e^-5^. The KEGG annotation was conducted using Diamond (http://www.diamondsearch.org/index.php, version 0.8.35) against the Kyoto Encyclopedia of Genes and Genomes database (http://www.genome.jp/keeg/) with an e-value cutoff of 1e^-5^.

Supplementary figure 1 Lefse analysis. Significant biomarker taxon of bacterial, fungal, and archaea communities in control group, potato intercropping, and soybean intercropping. In non-rhizosphere soils (a) bacterial community, (b) fungal community, and (c) archaea community. (d)Bacterial community and (e) fungal community in rhizosphere soil. In root (f) bacterial community, (g) eukaryotic community, and (h) archaea community. The three treatments were control group (PS_C/RS_C/R_C), potato intercropping (PS_P/RS_P/R_P) and soybean intercropping (PS_S /RS_S /R_S).


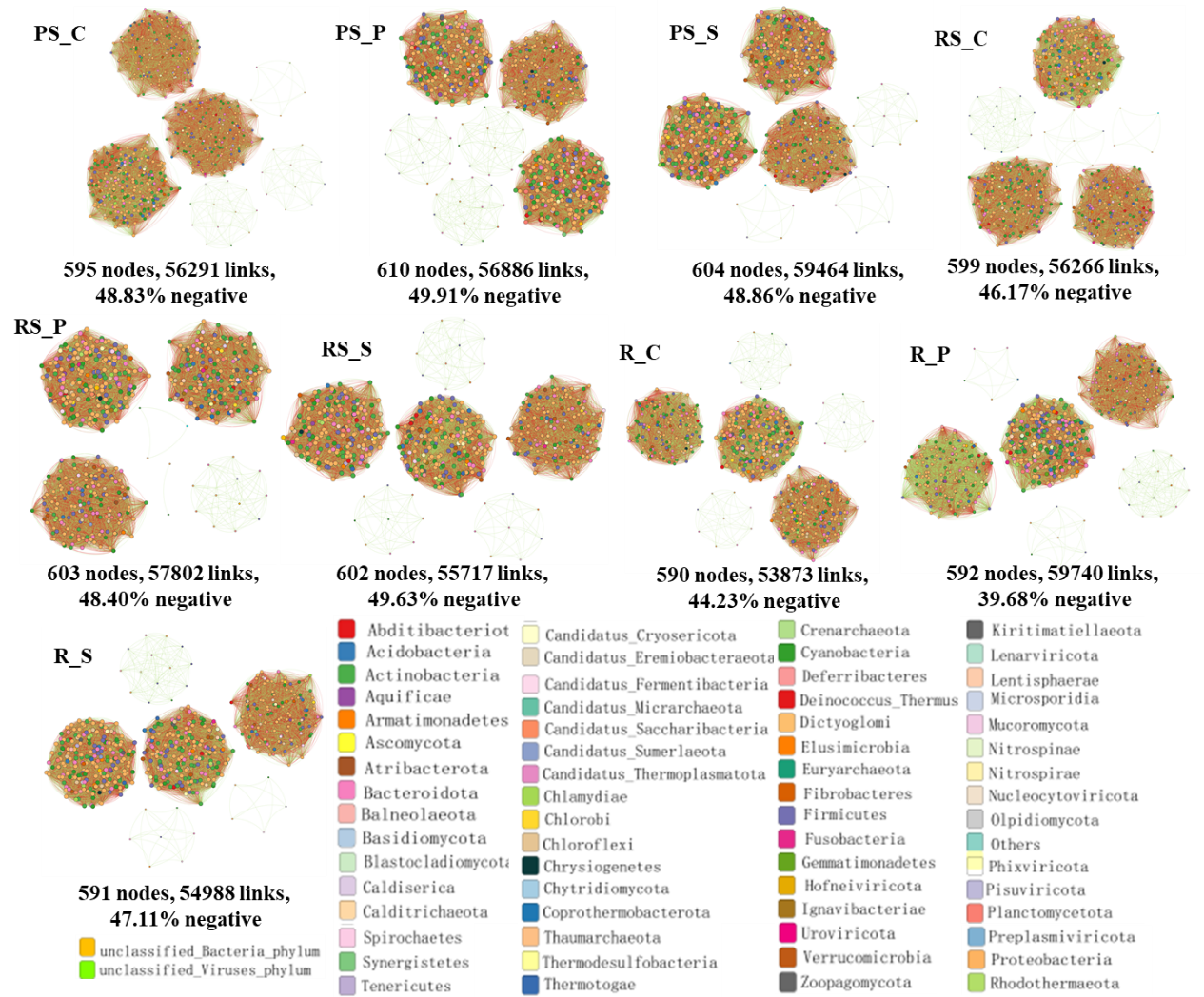


Supplementary figure 2 Co-occurrence network of bacteria in non-rhizosphere soil, rhizosphere soil and roots. The size of the nodes in the graph represents the abundance of the species, where the larger the abundance value, the larger the node. The color of the line represents a positive and negative correlation. Red indicates a positive correlation between species, while green indicates a negative correlation between species. The thickness of the line represents the magnitude of the correlation coefficient value. A thick line indicates a high correlation between species. A large number of lines indicates a close relationship between this species and other species. The three treatments were control group (PS_C/RS_C/R_C), potato intercropping (PS_P/RS_P/R_P) and soybean intercropping (PS_S /RS_S /R_S).


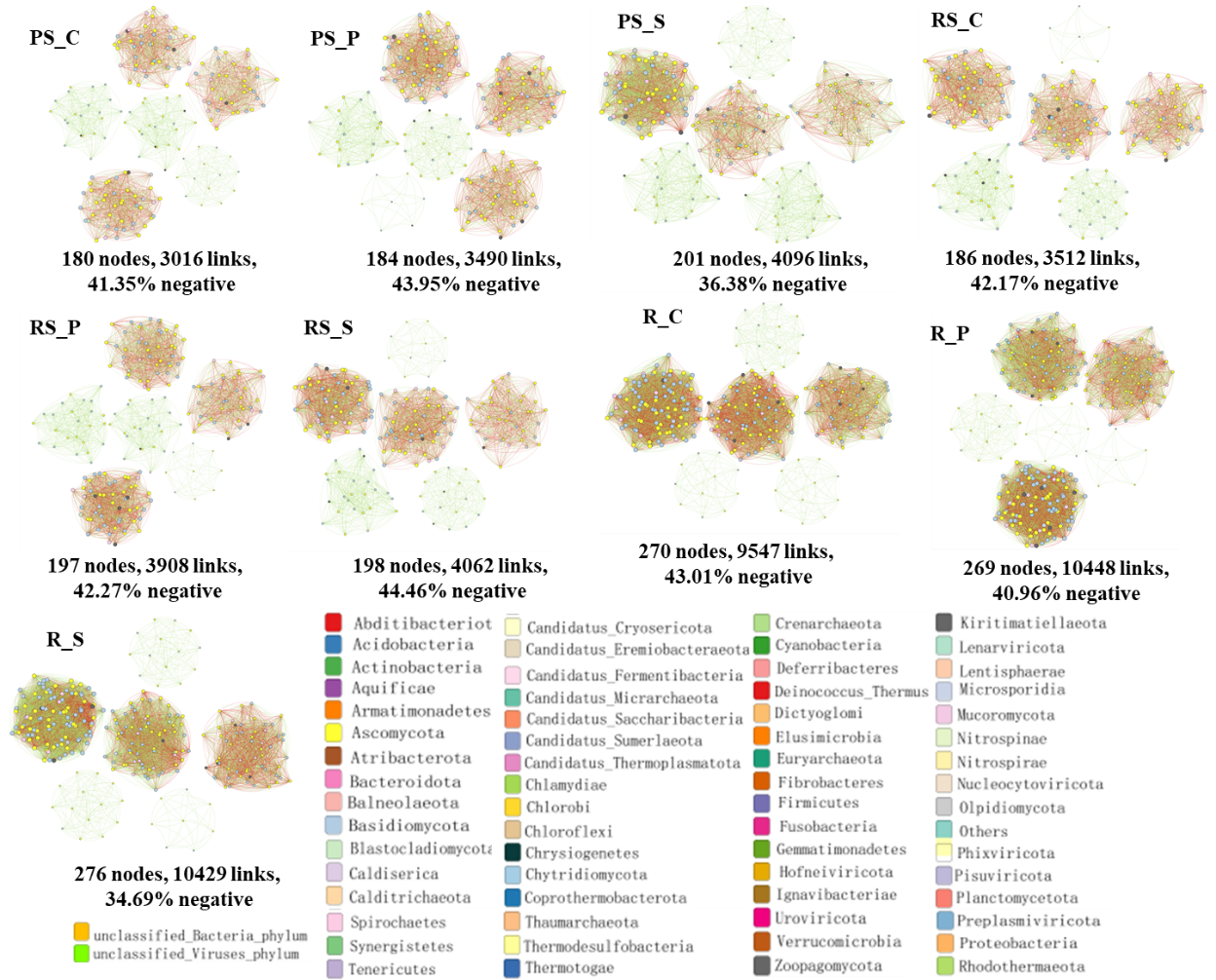


Supplementary figure 3 Co-occurrence network of eukaryote in non-rhizosphere soil, rhizosphere soil and roots. The size of the nodes in the graph represents the abundance of the species, where the larger the abundance value, the larger the node. The color of the line represents a positive and negative correlation. Red indicates a positive correlation between species, while green indicates a negative correlation between species. The thickness of the line represents the magnitude of the correlation coefficient value. A thick line indicates a high correlation between species. A large number of lines indicates a close relationship between this species and other species. The three treatments were control group (PS_C/RS_C/R_C), potato intercropping (PS_P/RS_P/R_P) and soybean intercropping (PS_S /RS_S /R_S).


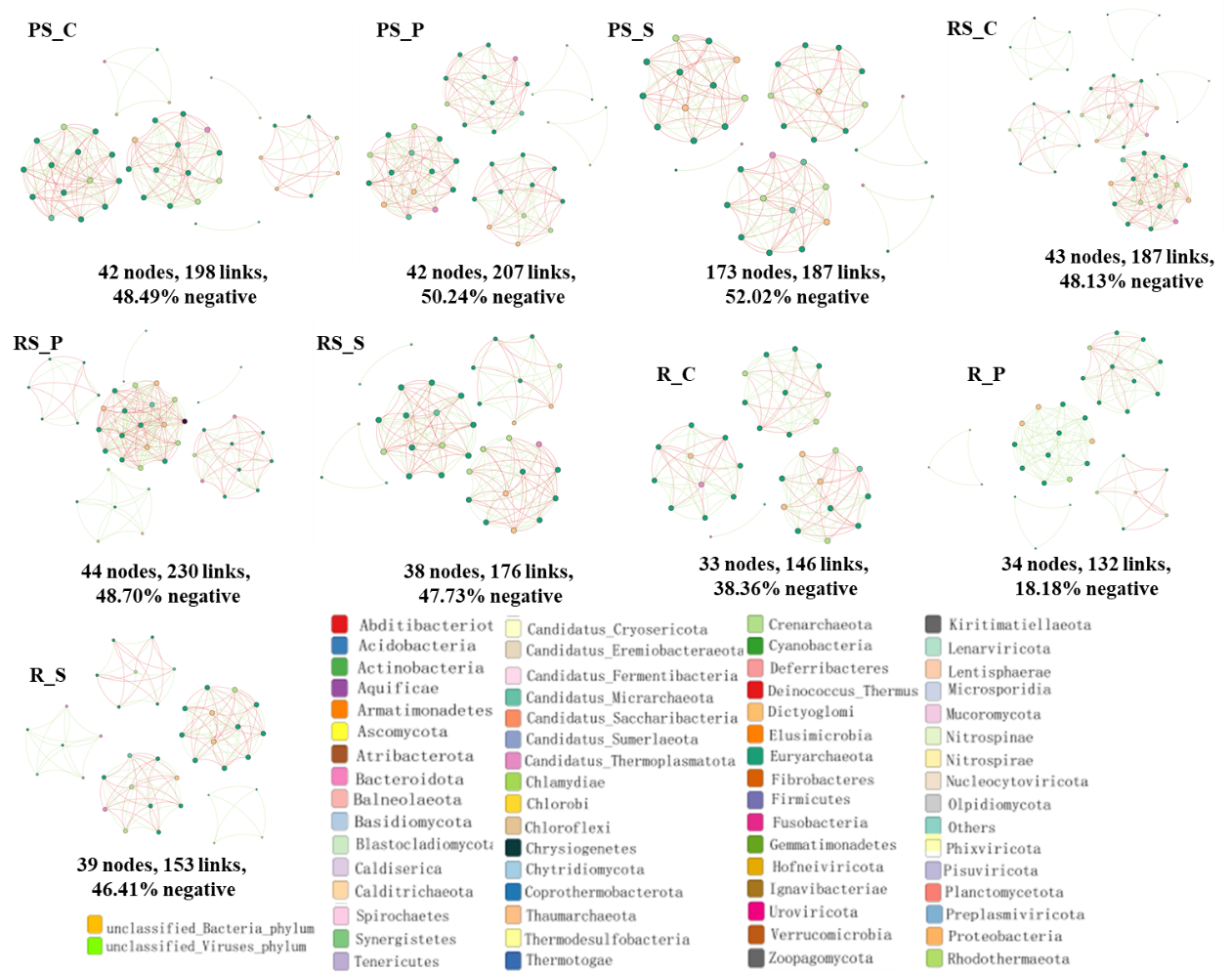


Supplementary figure 4 Co-occurrence network of archaea in non-rhizosphere soil, rhizosphere soil and roots. The size of the nodes in the graph represents the abundance of the species, where the larger the abundance value, the larger the node. The color of the line represents a positive and negative correlation. Red indicates a positive correlation between species, while green indicates a negative correlation between species. The thickness of the line represents the magnitude of the correlation coefficient value. A thick line indicates a high correlation between species. A large number of lines indicates a close relationship between this species and other species. The three treatments were control group (PS_C/RS_C/R_C), potato intercropping (PS_P/RS_P/R_P) and soybean intercropping (PS_S /RS_S /R_S).

Supplementary figure 5 COG and KEGG functional annotations of microorganisms in non-rhizosphere soils, rhizosphere soils, and roots. The three treatments were control group (PS_C/RS_C/R_C), potato intercropping (PS_P/RS_P/R_P) and soybean intercropping (PS_S /RS_S /R_S).

Supplementary figure 6 Changes of total abundance of genes related to nutrient metabolism and transport pathways in microbial communities. (a) Carbon decomposition, (b) Carbon fixation，(c) Nitrogen cycling, (d) Phosphorus cycling, and (e) Sulfur cycling。The three treatments were control group (PS_C/RS_C/R_C), potato intercropping (PS_P/RS_P/R_P) and soybean intercropping (PS_S /RS_S /R_S).

Supplementary figure 7 Species and functional contributions of genus (a) and phylum level (b) in rhizosphere soils. F1 Carbon metabolism，F2 ABC transporters，F3 Oxidative phosphorylation，F4 Pyruvate metabolism，F5 Glycolysis / Gluconeogenesis，F6 TCA cycle，F7 Pentose phosphate pathway，F8 Sulfur metabolism，F9 Nitrogen metabolism. The three treatments were control group (RS_C), potato intercropping (RS_P) and soybean intercropping (RS_S).

Supplementary figure 8 Species and functional contributions of genus (a) and phylum level (b) in root. F1 Carbon metabolism，F2 ABC transporters，F3 Oxidative phosphorylation，F4 Pyruvate metabolism，F5 Glycolysis / Gluconeogenesis，F6 TCA cycle，F7 Pentose phosphate pathway，F8 Sulfur metabolism，F9 Nitrogen metabolism. The three treatments were control group (R_C), potato intercropping (R_P) and soybean intercropping (R_S).

Supplementary table 1 Topological parameters of microbial communities

| Group | Total nodes | Total links | Average degree (avgK) | Average clustering coefficient (avgCC) | Average path distance (GD) | Modularity |
| --- | --- | --- | --- | --- | --- | --- |
| PS_C | 848 | 98020 | 231.18 | 1 | 1 | 0.64 |
| PS_P | 870 | 103864 | 238.78 | 1 | 1 | 0.66 |
| PS_S | 882 | 110212 | 249.91 | 1 | 1 | 0.62 |
| RS_C | 864 | 101891 | 235.86 | 1 | 1 | 0.65 |
| RS_P | 879 | 106844 | 243.10 | 1 | 1 | 0.66 |
| RS_S | 872 | 104304 | 239.23 | 1 | 1 | 0.65 |
| R_C | 937 | 125339 | 267.53 | 1 | 1 | 0.65 |
| R_P | 940 | 137664 | 292.90 | 1 | 1 | 0.54 |
| R_S | 951 | 131402 | 276.35 | 1 | 1 | 0.63 |

Supplementary table 2 Topological parameters of bacterial communities

| Group | Total nodes | Total links | Average degree (avgK) | Average clustering coefficient (avgCC) | Average path distance (GD) | Modularity |
| --- | --- | --- | --- | --- | --- | --- |
| PS_C | 595 | 56290 | 189.21 | 1 | 1 | 0.60 |
| PS_P | 610 | 56885 | 186.51 | 1 | 1 | 0.63 |
| PS_S | 604 | 59462 | 196.89 | 1 | 1 | 0.62 |
| RS_C | 599 | 56266 | 187.87 | 1 | 1 | 0.64 |
| RS_P | 603 | 57802 | 191.72 | 1 | 1 | 0.65 |
| RS_S | 602 | 55717 | 185.11 | 1 | 1 | 0.65 |
| R_C | 590 | 53873 | 182.62 | 1 | 1 | 0.63 |
| R_P | 592 | 59740 | 201.82 | 1 | 1 | 0.51 |
| R_S | 591 | 54986 | 186.08 | 1 | 1 | 0.63 |

Supplementary table 3 Topological parameters of eukaryote communities

| Group | Total nodes | Total links | Average degree (avgK) | Average clustering coefficient (avgCC) | Average path distance (GD) | Modularity |
| --- | --- | --- | --- | --- | --- | --- |
| PS_C | 180 | 3016 | 33.51 | 1 | 1 | 0.76 |
| PS_P | 184 | 3490 | 37.94 | 1 | 1 | 0.73 |
| PS_S | 201 | 4096 | 40.76 | 1 | 1 | 0.68 |
| RS_C | 186 | 3512 | 37.76 | 1 | 1 | 0.74 |
| RS_P | 197 | 3908 | 39.68 | 1 | 1 | 0.73 |
| RS_S | 198 | 4062 | 41.03 | 1 | 1 | 0.71 |
| R_C | 270 | 9547 | 70.72 | 1 | 1 | 0.63 |
| R_P | 269 | 10448 | 77.68 | 1 | 1 | 0.58 |
| R_S | 276 | 10429 | 75.57 | 1 | 1 | 0.58 |

Supplementary table 4 Topological parameters of archaea communities

| Group | Total nodes | Total links | Average degree (avgK) | Average clustering coefficient (avgCC) | Average path distance (GD) | Modularity |
| --- | --- | --- | --- | --- | --- | --- |
| PS_C | 42 | 198 | 9.43 | 1 | 1 | 0.62 |
| PS_P | 42 | 207 | 9.86 | 1 | 1 | 0.67 |
| PS_S | 41 | 173 | 8.44 | 1 | 1 | 0.69 |
| RS_C | 43 | 187 | 8.70 | 1 | 1 | 0.61 |
| RS _P | 44 | 230 | 10.46 | 1 | 1 | 0.51 |
| RS_S | 38 | 176 | 9.26 | 1 | 1 | 0.64 |
| R_C | 33 | 146 | 8.85 | 1 | 1 | 0.67 |
| R_P | 34 | 132 | 7.77 | 1 | 1 | 0.62 |
| R_S | 39 | 153 | 7.85 | 1 | 1 | 0.70 |

Supplementary table 5 Growth indicators of plants

|  | Ground diameter (m) | Tree height (m) | Coefficient of variation of tree height (%) | Coefficient of variation of ground diameter (%) |
| --- | --- | --- | --- | --- |
| Control | 0.014±0.003a | 2.44±0.25a | 10.25 | 19.25 |
| Potatoes-poplar | 0.013±0.003a | 2.40±0.26a |  |  |
| Soybean-poplar | 0.013±0.002a | 2.48±0.23a |  |  |

Supplementary table 6 Significance analysis of Chao index

|  | F-value | P-value |
| --- | --- | --- |
| Non-rhizosphere soil | 3163401.000 | 0.001 |
| Rhizosphere soil | 422800.000 | 0.001 |
| Root | 278.714 | 0.001 |

Supplementary table 7 Significance analysis of Shannon index

|  | F-value | P-value |
| --- | --- | --- |
| Non-rhizosphere soil | 16.125 | 0.004 |
| Rhizosphere soil | 0.765 | 0.506 |
| Root | 0.368 | 0.706 |

Supplementary table 8 Significance analysis of Simpson index

|  | F-value | P-value |
| --- | --- | --- |
| Non-rhizosphere soil | 2.400 | 0.171 |
| Rhizosphere soil | 4.429 | 0.066 |
| Root | 2.333 | 0.178 |

Supplementary table 9 Significance analysis of microbial community in non-rhizosphere soil

| Phylum | F-value | P-value |
| --- | --- | --- |
| *p__Proteobacteria* | 116444.846 | 0.001 |
| *p__Acidobacteria* | 14983.486 | 0.001 |
| *p__Actinobacteria* | 472124.333 | 0.001 |
| *p__Gemmatimonadetes* | 48100.200 | 0.001 |
| *p__Chloroflexi* | 863836.800 | 0.001 |
| *p__Verrucomicrobia* | 1062882.600 | 0.001 |
| *p__Bacteroidota* | 169909.717 | 0.001 |
| *p__Planctomycetota* | 20333.714 | 0.001 |
| *p__Thaumarchaeota* | 1468534.694 | 0.001 |

Supplementary table 10 Significance analysis of microbial community in rhizosphere soil

| Phylum | F-value | P-value |
| --- | --- | --- |
| *p__Proteobacteria* | 33712.000 | 0.001 |
| *p__Acidobacteria* | 16777.000 | 0.001 |
| *p__Actinobacteria* | 79117.000 | 0.001 |
| *p__Gemmatimonadetes* | 414.618 | 0.001 |
| *p__Chloroflexi* | 567.000 | 0.001 |
| *p__Verrucomicrobia* | 43.882 | 0.001 |
| *p__Bacteroidota* | 266.030 | 0.001 |
| *p__Planctomycetota* | 28.000 | 0.001 |
| *p__Thaumarchaeota* | 331.178 | 0.001 |

Supplementary table 11 Significance analysis of microbial community in root

| Phylum | F-value | P-value |
| --- | --- | --- |
| *p__Proteobacteria* | 3417821.348 | 0.001 |
| *p__Acidobacteria* | 190135.167 | 0.001 |
| *p__Actinobacteria* | 2115339.771 | 0.001 |
| *p__Gemmatimonadetes* | 1444.957 | 0.001 |
| *p__Chloroflexi* | 138.130 | 0.001 |
| *p__Verrucomicrobia* | 240042.463 | 0.001 |
| *p__Bacteroidota* | 16358.333 | 0.001 |
| *p__Planctomycetota* | 5231.250 | 0.001 |
| *p__Thaumarchaeota* | 7022.449 | 0.001 |

Supplementary table 12 Significance analysis of total nodes

|  | F-value | P-value |
| --- | --- | --- |
| Non-rhizosphere soil | 892.000 | 0.001 |
| Rhizosphere soil | 169.000 | 0.001 |
| Root | 163.000 | 0.001 |

Supplementary table 13 Significance analysis of links

|  | F-value | P-value |
| --- | --- | --- |
| Non-rhizosphere soil | 29531.928 | 0.001 |
| Rhizosphere soil | 1840.319 | 0.001 |
| Root | 11393.912 | 0.001 |

Supplementary table 14 Significance analysis of negative

|  | F-value | P-value |
| --- | --- | --- |
| Non-rhizosphere soil | 157.510 | 0.001 |
| Rhizosphere soil | 2339.735 | 0.001 |
| Root | 124579.000 | 0.001 |

Supplementary table 15 Significance analysis of nutrient content and enzyme activity in non-rhizosphere soil

|  | F-value | P-value |
| --- | --- | --- |
| Total phosphorus (g kg^-1^) | 0.61 | 0.574 |
| Organophosphorus (g kg^-1^) | 0.773 | 0.503 |
| Organic carbon (g kg^-1^) | 21.321 | 0.002 |
| Ammonium (mg kg^-1^) | 238.846 | 0.001 |
| Nitrate (mg kg^-1^) | 1701.211 | 0.001 |
| Total nitrogen % | 22.333 | 0.002 |
| Total carbon % | 126.843 | 0.001 |
| Leucine aminopeptidase (nmol h^-1^ g^-1^) | 1.047 | 0.407 |
| Urease (μg d^-1^g^-1^) | 171.896 | 0.001 |

Supplementary table 16 Significance analysis of nutrient content and enzyme activity of rhizosphere soil

|  | F-value | P-value |
| --- | --- | --- |
| Total phosphorus (g kg^-1^) | 0.182 | 0.838 |
| Organophosphorus (g kg^-1^) | 2.402 | 0.171 |
| Organic carbon (g kg^-1^) | 42.181 | 0.001 |
| Ammonium (mg kg^-1^) | 134.446 | 0.001 |
| Nitrate (mg kg^-1^) | 3508.174 | 0.001 |
| Total nitrogen % | 67.000 | 0.001 |
| Total carbon % | 58.235 | 0.001 |
| Leucine aminopeptidase (nmol h^-1^ g^-1^) | 20.995 | 0.002 |
| Urease (μg d^-1^g^-1^) | 11.830 | 0.008 |

Supplementary table 17 Significance analysis of nutrient content and enzyme activity of root

|  | F-value | P-value |
| --- | --- | --- |
| Total phosphorus (g kg^-1^) | 1028.770 | 0.001 |
| Total nitrogen % | 1179.500 | 0.001 |
| Total carbon % | 28.145 | 0.001 |
| Leucine aminopeptidase (nmol h^-1^ g^-1^) | 13.125 | 0.002 |
| Urease (μg d^-1^g^-1^) | 62.156 | 0.001 |
